# Human genetic research, race, ethnicity and the labeling of populations: recommendations based on an interdisciplinary workshop in Japan

**DOI:** 10.1186/1472-6939-15-33

**Published:** 2014-04-23

**Authors:** Yasuko Takezawa, Kazuto Kato, Hiroki Oota, Timothy Caulfield, Akihiro Fujimoto, Shunwa Honda, Naoyuki Kamatani, Shoji Kawamura, Kohei Kawashima, Ryosuke Kimura, Hiromi Matsumae, Ayako Saito, Patrick E Savage, Noriko Seguchi, Keiko Shimizu, Satoshi Terao, Yumi Yamaguchi-Kabata, Akira Yasukouchi, Minoru Yoneda, Katsushi Tokunaga

**Affiliations:** 1 Institute for Research in Humanities, Kyoto University, Yoshidahonmachi, Sakyo-ku, Kyoto 606-8501, Japan; 2 Department of Biomedical Ethics and Public Policy, Graduate School of Medicine, Osaka University, Suita, Japan; 3 Laboratory of Genome Anthropology, Department of Anatomy, Kitasato University School of Medicine, Sagamihara, Japan; 4 Faculty of Law and the School of Public Health, University of Alberta, Edmonton, Canada; 5 Laboratory of Medical Informatics, RIKEN Center for Genomic Medicine, Yokohama, Japan; 6 The Open University of Japan, Tokyo, Japan; 7 Institute of Data Analysis, StaGen Co.Ltd., Tokyo, Japan; 8 Department of Integrated Biosciences, Graduate School of Frontier Sciences, The University of Tokyo, Kashiwa, Japan; 9 Department of English Language and British and American Studies, Faculty of Humanities, Musashi University, Tokyo, Japan; 10 Department of Human Biology and Anatomy, Graduate School of Medicine, University of the Ryukyus, Nishihara, Japan; 11 Department of Art Studies, Meiji Gakuin University, Tokyo, Japan; 12 Department of Musicology, Tokyo University of the Arts, Tokyo, Japan; 13 Department of Environmental Changes, Graduate School of Social and Cultural Studies, Kyushu University, Fukuoka, Japan; 14 Department of Zoology, Faculty of Science, Okayama University of Science, Okayama, Japan; 15 Graduate School of Medicine, Kobe University, Kobe, Japan; 16 Department of Integrative Genomics, Tohoku Medical Megabank Organization, Tohoku University, Miyagi, Japan; 17 Department of Human Science, Faculty of Design, Kyushu University, Fukuoka, Japan; 18 Laboratory for Radiocarbon Dating, The University Museum, The University of Tokyo, Tokyo, Japan; 19 Department of Human Genetics, Graduate School of Medicine, The University of Tokyo, Tokyo, Japan

**Keywords:** Population descriptors, Ethics, Labeling, Race, Ethnicity, Japanese, Asian, Mongoloid

## Abstract

**Background:**

A challenge in human genome research is how to describe the populations being studied. The use of improper and/or imprecise terms has the potential to both generate and reinforce prejudices and to diminish the clinical value of the research. The issue of population descriptors has not attracted enough academic attention outside North America and Europe. In January 2012, we held a two-day workshop, the first of its kind in Japan, to engage in interdisciplinary dialogue between scholars in the humanities, social sciences, medical sciences, and genetics to begin an ongoing discussion of the social and ethical issues associated with population descriptors.

**Discussion:**

Through the interdisciplinary dialogue, we confirmed that the issue of race, ethnicity and genetic research has not been extensively discussed in certain Asian communities and other regions. We have found, for example, the continued use of the problematic term, “Mongoloid” or continental terms such as “European,” “African,” and “Asian,” as population descriptors in genetic studies. We, therefore, introduce guidelines for reporting human genetic studies aimed at scientists and researchers in these regions.

**Conclusion:**

We need to anticipate the various potential social and ethical problems entailed in population descriptors. Scientists have a social responsibility to convey their research findings outside of their communities as accurately as possible, and to consider how the public may perceive and respond to the descriptors that appear in research papers and media articles.

## Background

With the rapid technical advances that have occurred in genome research, human genetic samples can now be analyzed on a massive scale and at an unprecedented speed. It is likely only a matter of time before this avalanche of genomic information is harnessed to allow healthcare decisions, such as the use of pharmaceuticals and the stratification of treatment protocols, to be increasingly tailored in a manner that will be informed by individual genetic predispositions. There are, of course, many social and ethical issues involved in human genome research and in the application of the emerging knowledge [1,2]. One of the important, yet potentially complex, issues is how best to describe and report the populations that are being studied in the exploration of genetic variations [3]. There is concern that the use of improper and/or imprecise terminology, particularly language tied to concepts of “race” and “ethnic group” has the potential to both generate and reinforce racial and ethnic prejudices and diminish the clinical value of relevant research, as the massive literature shows [4,5]. In addition, broader terms such as “continental ancestry group” may not satisfactorily capture population differentiation on a sub-continental scale [6].

The issue of population descriptors has attracted a good deal of academic attention in North America and some European communities [7]. In these regions, there is much sensitivity to the ways populations are described. This is likely due, in part, to the history of racism within biomedical research and growing social awareness of the significance of ethnic and racial issues.

In contrast, many other regions, including Japan and some other regions in Asia, where the myth of raceless society have long persisted, have failed to tackle the topic, resulting in population descriptors sometimes being overgeneralized and ethically problematic. The number of genomic studies is skyrocketing in many countries and it is urgent that researchers based outside Europe and North America take a more active role in addressing the issues associated with the use and misuse of population descriptors.

Recognizing the importance of addressing these issues, we held a two-day workshop on January 7-8^th^, 2012, in Tokyo, which brought together scholars in diverse fields including those in the humanities, social sciences, medical sciences and genetics. The scientists shared their actual practices and relevant experiences on the use of population descriptors in publications and communications including review processes, while researchers in the humanities and social sciences discussed racism in the past and contemporary social issues involving minority groups. Although our focus was on the social and ethical issues involving Asian communities, in particular Japan, we hoped that our general conclusions would apply to other countries and communities with similar situations. At the end of the event, we agreed to produce a set of guidelines for reporting genetic studies involving populations in Asia, based in part on the recommendations of Caulfield et al. [8]. With this conference as the start, the discussion continued after the workshop through various methods such as core authors’ meetings, e-mail communications, and video conferences. In this article, we report on the substance of these discussions focused on population descriptors and present the recommendations primarily targeting the genetics researchers in the region.

## Discussion

### Ethical and social issues arising from genetic research involving human populations

Research findings are often represented with over generalized descriptors such as “Asian” or other continental terms. In reality, samples are taken from much more discrete groups or specific and identifiable geographical regions. This tendency may be the result of a number of forces. Researchers sometimes attempt to draw more general conclusions than the actual data can support. In other cases, researchers may feel that without broad terms, it is difficult to gain recognition in the review process for publication or for the obtainment of a grant. The lack of education and training for scientists and researchers regarding the use of descriptors and associated problems seems to be another cause of overgeneralization. Indeed, at the workshop, some of the participants shared their experiences of receiving such pressure to generalize from both research institutions and publishers. At the current time there is no data that maps the extent of this phenomenon and, as such both quantitative and qualitative systematic investigation is needed. The following examples demonstrate how, from the perspective of genetic research done in Asia, the inappropriate use of population descriptors could cause confusion and social controversies.

Although the term “Mongoloid” is rarely used today in North America and Europe, the situation is different in Japan and some other regions of Asia. Our preliminary analysis showed 113 hits in *PubMed* that contain the term “Mongoloid” in titles or abstracts of papers published during the period of 2004-2013, with no signs that use is decreasing. However, even among researchers, there is little awareness of the issues and little consistency in use, and its meaning can vary significantly depending on context [9-11]. Some researchers may use the term to designate a population in a particular or a variety of regions, including Eastern Asia, Southeast Asia or indigenous peoples in North America [12]. For others, it refers only to East Asians, or may be a synonym for the more generic Asian [13,14]. Moreover, the term has, in the past, been used to refer to individuals with Down’s syndrome. In general, despite its continuing use, the term is problematic both because of the uncertainty regarding the population referred to, and because of its past controversial use [15,16].

Another example of the challenges associated with the use of population descriptors can be found in the frequent use of the terms European, African, and Asian. These continental terms are tremendously broad in scope. At the Tokyo meeting, for example, it was noted that even among the Japanese researchers, there was no unitary understanding of what populations should be considered “Asian.”

More importantly, these terms can, in some contexts, be interpreted as referring to white, black, and Asian, the three classic, and socially constructed “races.” There continues to be a great deal of academic work that highlights the degree to which these broad “racial” categories are, in reality, social constructs [17-19]. Although we should not overlook the correlation between “race” and socio-economic inequality involving factors such as health care and medical care, such discussion has usually arisen within the context of some North American and European societies. However, outside of these societies, the divergence between samples and population descriptors is also problematic. When the actual samples in the name of “European”, “African”, and “Asian” are taken from certain limited groups, without taking into account significant diversity within each region, it is unlikely that such broad terms have any scientific meaning, at least from the perspective of genetics on the global level [20,21]. Moreover, the research results may be taken as supporting the classic “racial” categories, with any discovered “differences” misinterpreted as genetically determined “racial differences.”

The importance of the distinction between race and ethnicity cannot be overemphasized as the latter pays close attention to (presumably) shared cultural factors such as language, diet, and religion [22]. When considering the contribution of environmental as well as genetic factors to diversity within each continental region, the scientific validity of the use of such broad terms to describe samples becomes even more questionable.

In contrast to the above tendency to prefer broad terms, an influential study based on genome-wide 50 K SNP data reveals the detailed patterns of genetic differentiations within “Asians” [23]. The genetic ancestry of most populations was associated with ethnic and linguistic affiliations. Along the same lines, an analysis of 7,003 individuals from across Japan reveals interesting regional variations within the “Japanese” population. At one level, most Japanese fell into two main clusters from individuals taken in mainland Japan and those in Okinawa in a principal component analysis (PCA) plot based on genome-wide 140 K SNP data. Upon closer look, even among mainland Japanese, statistically meaningful genetic differentiation was found among individuals in different regions, such as Tohoku, Kanto, Kinki, and Kyushu [24].

The above study highlights that even populations traditionally presumed to have a high degree of homogeneity may have local genetic differentiations, that make the use of broader population terms less scientifically or clinically relevant. Researchers should strive to select terms that, as much as possible, reflect the sample population and nature of each study. Since genetic subpopulation structure is still generally unknown, sampling without considering the specifics of the subject population could cause false positive results on risk alleles of diseases. In addition, differences in whole genome sequences between individuals belonging to different populations should not be overgeneralized and misinterpreted as population differences.

Through our dialogue, it became apparent that the ways in which descriptors are selected sometimes differ depending on specialized fields. For example, researchers in physical/biological anthropological studies have a relatively long history of working on population genetics studies concerning local residents from whom they obtain sample data, and accumulate information on various populations from the perspective of long-term human evolution. Medical studies, on the other hand, are more concerned with the applicability of genetic studies contributing to the diagnosis and treatment of diseases. Disease gene surveys often take samples from patients at hospitals without controlling such factors as current location of residence or generational continuity in each place. Such disciplinary differences in research purposes and methods have sometimes created different understandings and placed varying levels of attention on the issue of population description. This is one example why dialogue between scholars in different disciplines is indispensable in considering appropriate population descriptors.

There has been a growing discussion of the “co-production” of knowledge by the interplay between science and society [25]. The popular press is often blamed for the use of inappropriate or imprecise terms in the context of population genetic studies, whereas many scientists may believe that they take adequate precautions when describing the study samples, defining populations, and presenting discussions based on their research results. However, evidence indicates that imprecise and less than ideal descriptors are introduced throughout the research communication process [26]. If these descriptors are not carefully chosen, they create the potential for confusion both within the scientific community and in the wider society, leading to research inefficiencies and various social, ethical, and clinical problems [7].

What, then, would be a more desirable way to describe populations under study? The key is to use population descriptors that are scientifically valid for the particular study. For the first step, we recommend the use of population descriptors with more specific characteristics, such as geographical location and ethnic labeling as previously attempted – albeit imperfectly [27] – by research initiatives like the International HapMap Project [28]. This recommendation is based on the fact that various studies demonstrate the strong correlation between genetic distances and distances based on geography as well as ethnic affiliations [23,29]. This is, we believe, a better solution, but not a final one. Even when scientists choose more specific terminology, they have to explain the rationale behind the descriptors and what rules they employ in selecting the samples and defining the population.

Finally, the importance of education for undergraduate and graduate students as well as young trainees in human genetics and medicine cannot be overemphasized. It is urgent to prepare appropriate curriculums incorporating these ethical and social issues in order to effectively change the awareness of scholars and practitioners in the near future.

## Recommendations

Based on the discussions and analyses described in the previous section, we have come up with the following nine recommendations.

1. In selecting descriptors, use specific names for populations or groups of people closely reflecting the make-up of the sample, while protecting the privacy of individuals included.

2. When using ethnological information for labeling, respect the cultural sensitivities of the populations and employ names that correspond to their cultural and ethnic backgrounds as much as possible. If not, clarify the definition of the names of populations used in the study, and explain why such descriptors have been chosen.

3. Explain how, where, and when sample data are collected, and who the concerned individuals are donors–as long as the information does not impinge on individual privacy. Also, the description of sampling date is important because allele frequencies could change in a population owing to demography, migration, and drift in a short time span.

4. Avoid overly broad category names such as Asian, European, or African. Recognize that the use of such names without scientific justification could cause confusion, misinterpretation, and social controversy - particularly if the research results are interpreted in a manner that could emphasize the existence of these “racial” categories. If the use of broad category names is necessary, there must be sufficient scientific grounds and explanation.

5. When genetic differences are ones in degrees of frequencies among different populations, avoid typological discussions and emphasize that the differences are a matter of frequency and probability, not differences that are clear-cut and discrete.

6. Be alert to the possibility that research on human populations could cause various kinds of social and ethical problems; therefore, endeavor to take steps to anticipate relevant issues that may emerge, including seeking to collaborate, as appropriate, with colleagues in other relevant disciplines (e.g., medical researchers and researchers in the humanities and social sciences). It is also important to recognize that scientific activities are also influenced by social and political factors. Population descriptors are no exception.

7. Prepare an easily understandable summary of research findings, so that reporters for newspapers and other popular media can prepare proper reports that utilize appropriate population descriptors.

8. Point out any mistakes or misinterpretations of the research results after the reports are released to the public, and if opportunity allows, confirm them before public release.

9. Incorporate the above considerations into the education curriculums for emerging trainees at an early stage in their careers [8].

## Summary

In this age of genomics, differences between populations are often reported as having genetic bases [26]. However, misunderstanding and extended interpretation of the results might contribute to discrimination, or justify health care and socio-economic inequalities [30]. Therefore, we need to anticipate the various potential social and ethical problems associated with population descriptors. Scientists have a social responsibility to convey their research findings outside of their communities as accurately as possible, and to consider how the public may perceive and respond to the descriptors that appear in research papers and media articles. Researchers in the humanities and social sciences may be able to contribute to the identification of potential social and ethical problems involving population descriptors. As such, there is a compelling need for truly interdisciplinary dialogue and collaboration between professionals in genetics, medical science, the humanities, and social sciences. We believe that such activities are particularly needed in the countries and communities outside of North America and Europe, where the issues of race, ethnicity and genetic research are not discussed extensively.

What we have discussed is merely a first step in the process of addressing the challenges associated with the use of population descriptors in the context of genetic research, and we hope that it will encourage action and the exchange of ideas and opinions, especially among the relevant research communities in Japan and other Asian countries.

## Competing interests

The authors declare that they have no competing interests.

## Authors’ contributions

YT, KK^2^, HO conceived the idea of the research and organized the workshop in Tokyo in 2012. YT, KK, HO, TC and KT wrote the core of the manuscript. TC, NK, AF, NS, YYK, KT made presentations at the conference. HM conducted the preliminary analyses for the discussion. SH, SK, KK^9^, RK, AS, PES, KS, ST, AY, MY contributed to discussion at the workshop and subsequently gave comments on the drafts of the manuscript. All authors read and approved the final manuscript.

## Pre-publication history

The pre-publication history for this paper can be accessed here:

http://www.biomedcentral.com/1472-6939/15/33/prepub
